# The future is now: our experience starting a remote clinical trial during the beginning of the COVID-19 pandemic

**DOI:** 10.1186/s13063-021-05537-6

**Published:** 2021-09-07

**Authors:** Hans H. Liu, Michael D. Ezekowitz, Michele Columbo, Oneib Khan, Jack Martin, Judith Spahr, David Yaron, Lisa Cushinotto, Luciano Kapelusznik

**Affiliations:** 1 Department of Medicine, Bryn Mawr Hospital, Bryn Mawr, PA USA; 2 The Sidney Kimmel Medical College, Philadelphia, PA USA; 3 Bala Cynwyd, PA USA; 4 Lankenau Internal Medicine Residency Program, Lankenau Hospital, Main Line Health System, Wynnewood, PA USA; 5 Lewes, DE USA; 6 Bryn Mawr Family Practice Residency, Bryn Mawr Hospital, Bryn Mawr, PA USA; 7 Department of Pharmacy, Bryn Mawr Hospital, Bryn Mawr, PA USA

**Keywords:** Prospective clinical trial, SARS-CoV-2, COVID-19 pandemic, Hydroxychloroquine, Remote ECG monitoring, Trial management

## Abstract

**Background:**

The World Health Organization declared the outbreak of SARS-CoV-2 a pandemic on February 11, 2020. This organism causes COVID-19 disease and the rapid rise in cases and geographic spread strained healthcare systems. Clinical research trials were hindered by infection control measures discouraging physical contact and diversion of resources to meet emergent requirements. The need for effective treatment and prevention of COVID-19 prompted an untested investigational response. Trial groups adapted approaches using remote enrolment and consenting, newly developed diagnostic tests, delivery of study medications and devices to participants’ homes, and remote monitoring to ensure investigator/enrollee safety while preserving ethical integrity, confidentiality, and data accuracy.

**Methods:**

Clinical researchers at our community health system in the USA undertook an outpatient randomized open-label study of hydroxychloroquine (HCQ) prophylaxis versus observation of SARS-CoV-2 infection in household COVID-19 contacts. Designed in March 2020, challenges included COVID-19 infection in the research group, HCQ shortage, and lack of well-established home SARS-CoV-2 tests and remote ECG monitoring protocols in populations naive to these procedures. The study was written, funded, and received ethical committee approval in 4 months and was completed by September 2020 during a period of fluctuating infection rates and conflicting political opinions on HCQ use; results have been published. Singular methodology included the use of a new RNA PCR saliva SARS-CoV-2 home diagnostic test and a remote smartphone-based 6-lead ECG recording system.

**Results:**

Of 483 households contacted regarding trial participation, 209 (43.3%) did not respond to telephone calls/e-mails and 90 (18.6%) declined; others were not eligible by inclusion or exclusion criteria. Ultimately, 54 individuals were enrolled and 42 completed the study. Numbers were too small to determine the efficacy of HCQ prophylaxis. No serious treatment-related adverse events were encountered.

**Conclusions:**

Flexibility in design, a multidisciplinary research team, prompt cooperation among research, funding, ethics review groups, and finding innovative study approaches enabled this work. Concerns were balancing study recruitment against unduly influencing individuals anxious for protection from the pandemic and exclusion of groups based on lack of Internet access and technology. An issue to address going forward is establishing research cooperation across community health systems before emergencies develop.

**Trial registration:**

ClinicalTrials.govNCT04652648. Registered on December 3, 2020.

## Background

A prospective, randomized clinical trial of hydroxychloroquine (HCQ) prophylaxis of SARS-CoV-2 acquisition by household contacts of COVID-19 cases in suburban Pennsylvania, USA, was designed and conducted between March and September 2020 and has been published [1]. Some challenges in rapidly completing this study were prevalent in the USA during this time frame and our experience highlights pitfalls and lessons potentially applicable to future trials.

### Onset of the COVID-19 pandemic

The COVID-19 pandemic posed challenges to healthcare systems worldwide on a scale that has not been encountered in generations. The rapid rise in infections with significant morbidity and mortality coupled with extensive geographic spread strained medical professionals and healthcare systems to the breaking point. Human populations proved highly susceptible to SARS-CoV-2 infection, but routes of transmission were not fully understood initially and there were no rapid tests for detection nor proven agents for treatment or prevention. On January 30, 2020, the World Health Organization (WHO) declared the coronavirus outbreak to be a public health emergency of international concern [2]. This was followed by a WHO pandemic declaration on February 11, 2020, and on March 11, 2020, the disease process was designated COVID-19 [2].

### Proposed agents for treatment and prevention of COVID-19

During spring 2020, reports indicated COVID-19 was contagious via respiratory droplets [3] with higher secondary household attack rates than the coronaviruses causing SARS or MERS [4]. The median incubation period of COVID-19 was estimated to be 5 days [5]. The secondary infection rate in contacts of active COVID-19 cases in Shenzhen, China, from January 14 to February 12, 2020, was reported as 11.2% on April 27, 2020 [6]. A later worldwide survey found a wide range of secondary infection rates ranging from 4.6 to 49.56% [7]. An even higher rate of 53% was reported in COVID-19 household contacts from the USA between April and September 2020 [8]. Significantly, many secondary cases have been asymptomatic [9]. Efforts to prevent the spread of coronavirus SARS-CoV-2 utilized physical distancing and personal protective equipment (PPE). Use of both well-known and investigational pharmacologic agents to treat and/or prevent coronavirus infections has been proposed in the past [10]. One such agent was hydroxychloroquine (HCQ) used for decades to treat rheumatologic diseases and prophylax against malaria [11]. In vitro data suggested HCQ had activity against SARS-CoV-2 infection [12, 13] possibly via interference with viral membrane fusion and entry into mammalian cells [13]. The US Food and Drug Administration (FDA) issued an emergency use authorization (EUA) for chloroquine and hydroxychloroquine to treat COVID-19 in hospitalized patients outside of clinical trials on March 28, 2020 [14]. Treatment of asymptomatic healthcare workers and household contacts of COVID-19 infections with HCQ was recommended by an advisory from the National Task Force for COVID-19, Indian Council of Medical Research, on March 21, 2020 [15]. However, healthcare systems in the USA were not given specific guidance about prophylactic regimens against SARS-CoV-2.

### Local goals and evolving clinical trial design

The Main Line Health System (MLHS) is a community teaching hospital system of four acute care hospitals with 1058 beds including 138 in intensive care units. MLHS began to see COVID-19 cases at the beginning of March 2020, and by the end of that month, an average of 20 suspected or proven COVID-19 cases were being admitted daily. Table 1 shows that initially MLHS admissions roughly paralleled local, regional, and national COVID-19 new cases.

**Table 1:** Average daily COVID-19 cases locally, regionally, and nationally during 2020

Location	March 31	April 15	June 1	Sept 15
MLHS hospital admissions^a^ (capacity 1058 beds)	20	42	13	2
Montgomery County^b^ (population 830,000)	37.6	116.5	77.1	42.9
Pennsylvania State^c^ (population 13,002,700)	570	1464	569	836
USA^c^ (population 331,002,651)	20,350	28,083	21,314	39,000

^a^Daily COVID-19 patient census [16]; ^b^14-day average daily hospitalized and outpatient cases [17]; ^c^7-day average daily hospitalized and outpatient cases [18]

Conducting a local study of HCQ prophylaxis of household contacts of individuals with COVID-19 was proposed by a practicing infectious disease physician in early March 2020. A second infectious disease physician researched available literature and input was sought from other healthcare providers including a hospital pharmacist specializing in infectious diseases and antimicrobial stewardship and a practicing allergist with extensive experience serving on institutional review boards. Discussions were carried out by telephone and online as in-person meetings had been severely restricted by the health system. An initial barrier to conducting a clinical trial was a shortage of HCQ. This was due to news reports of in vitro activity of HCQ against coronavirus and ensuing public demand despite the lack of any human trial data. The availability of study drugs was addressed when a manufacturer donated a supply of 200 mg tablets; one of the MLHS pharmacies agreed to manage storage and distribution. However, the large tablet labeled with the drug name precluded a blinded placebo-controlled study [19].

### Worldwide experience 1 year into the COVID-19 pandemic

Now more than 1 year into the pandemic, a number of publications have focused on issues impacting the planning and conduct of clinical trials in this setting [20]. Balancing clinical and research demands [21] and innovation and cooperation on studies have helped overcome many pandemic-associated barriers [22]. It has been suggested that pragmatic clinical trials may improve response times and improve trial efficacy during a global pandemic [23] and that multidisciplinary teams may have an advantage in adapting to new and changing conditions [24]. However, it is clear that the ethics of decision-making must be carefully preserved when a disease is not well-understood and yet there is enormous pressure for a rapid response [25, 26].

Among adjustments forced upon clinical research groups by the COVID-19 pandemic was the need to protect study participants and research staff from a highly contagious pathogen [27]. This applied to both non-COVID-19-related and COVID-19 research [28] and was especially true in fields focusing on face-to-face patient encounters. One survey noted that 26 of 30 US research sites involved in a skin disease trial had to suspend work [29] when COVID-19 developed. Procedure-oriented specialities such as surgery [30], breast imaging [31], and electroencephalography [32] and those prone to aerosol generation [33] also found clinical and research activity curtailed. Minimizing COVID-19 spread has led many healthcare practices to adopt videoconferencing with patients [20, 34] but this has had limitations. For example, even if the rate of patient follow-up visits was maintained, pediatric research units in Europe reported a marked decline in throat cultures obtained during the COVID-19 pandemic [35].

A July 2020 editorial [36] reminded clinical trial investigators to protect scientific integrity while still encouraging protocol adherence and enrolment retention; recommended were emphasizing proper use of PPE, building in adequate physical distancing, and administering therapies at participants’ homes when feasible. Electronic data collection was also encouraged; its sometimes inconsistent function was tolerated because “imperfect collection is more useful than no data” [36]. Developing integrated platforms for national and international sharing of COVID-19 pandemic data was also encouraged [37].

### Ethical issues in conducting COVID-19-related trials

Practical and ethical challenges encountered in clinical research during the COVID-19 pandemic included protocol deviations from canceled face-to-face study visits or missed laboratory specimen collection [38]. Reconciling this with the need to protect study participants and the public generated significant discussion [21] with “minimal risk” becoming more difficult to quantify [39]. Obtaining consumer input into the research design has also been problematic [40]; reasons included practical and official limitations to travel and meetings, changing understanding of COVID-19 pathogenesis and side effects of treatments, and conflicting public advice about participating in or avoiding certain activities, exposures, and medical therapies. Note is made of the confused state of the literature on these topics in spring 2020; much of the available information was from small studies, often observational and issued as preprints without peer review, which were then picked up by news services. Pre-existing inequities in healthcare services also contributed to exclusion of groups disproportionately affected by COVID-19 and/or underrepresented in healthcare research [41]; this was evident in the USA and elsewhere. Finally, the responsibility of academic medical centers and their research programs to healthcare workers in training was impacted [42] including loss of resident physician elective time due to overwhelming clinical demands.

## Clinical trial methodology

### Participant identification and enrolment

Potential study participants were identified by infectious disease consultants and medical resident physicians treating patients in MLHS acute care hospitals. These investigators asked inpatients with positive COVID-19 nasal PCR tests on admission for permission to contact their household members. Most individuals were amenable, though some declined permission. In cases where the infected individual felt they could not answer on behalf of their household and wanted to discuss participation with them first, study literature was provided and permission requested again later. This was acceptable to the MLHS institutional review board. Telephone calls were placed by the clinical trial study coordinator to home telephone numbers; in many cases, messages had to be left on voice mail. The large volume of COVID-19 cases early on, the need to contact potential enrollees promptly after exposure, limitations in study staff time, and the desire to avoid any appearance of pressuring potential participants generally limited attempted contact to one phone call. Study *inclusion criteria* were age ≥ 18 years, exposure to a COVID-19-infected individual in the same household within 5 days of diagnosis, ability to give informed consent to participate in a clinical study, ability to swallow oral medications, and access to a smartphone. *Exclusion criteria* included allergy or intolerance to HCQ (Plaquenil^R^), weight less than 85 pounds, eye disease affecting the retina, severe kidney or liver disease, G6PD deficiency, porphyria, long QTc ECG abnormality or family history of long QTc abnormality and other major ECG abnormalities, taking any of a list of medications that can affect the QT interval, current pregnancy or attempt to become pregnant, current hospitalization, symptomatic with fever or cough, or lack of access to a smartphone.

### At-home diagnostic testing and remote ECG monitoring

Eligible respondents agreeable to study enrolment and meeting inclusion criteria received informed consent forms online that were signed electronically. Randomization occurred prior to SARS-CoV-2 testing. The study protocol was an open-label trial; participants were randomized 2:1 to HCQ 200 mg orally twice a day for 10 days versus an observation group. All individuals within one household were randomized to the same group and followed for 14 days. The goal was a minimum of 170 enrollees over 2 months which was judged adequate to detect a relative reduction of infection rate from 50 to 25% in patients randomized to HCQ. Study medication, if applicable, was delivered to participants’ homes. A newly FDA-approved home collection SARS-CoV-2 saliva PCR test (Accurate Diagnostics Lab, South Plainfield, NJ, USA) was shipped to enrollees with instructions to return test samples in transport medium to a processing laboratory via prepaid shipping envelopes on day 1 and day 14. Study investigators contacted participants about possible COVID-19 symptoms at five time points during the study. Primary endpoints were the development of COVID-19 symptoms with a positive coronavirus PCR test by day 14, development of a positive coronavirus PCR test without symptoms by day 14, hospital admission by day 14, death by day 14, and EKG evidence of cardiotoxicity in both the HCQ group and the observation groups.

ECG monitoring software was downloaded by study enrollees to their smartphones and hardware-monitoring pads for attachment to the phones were shipped to all study households. Submission of daily ECG tracings by each participant during the study was requested. Details of the monitoring protocol and cardiac safety measures are reported in the publication presenting the study’s ECG findings [1].

## Results

### Local experience in organizing, funding, and conducting trial

The local threat of SARS-CoV-2 infection was realized when the researcher drafting the trial protocol contracted COVID-19 from non-occupation-related travel. Though hospitalization was avoided, the illness was debilitating. Fortunately, the almost completed draft was handed off online and the protocol discussed with a local foundation that supports clinical research in our health system as well as the MLHS institutional review board (IRB). The foundation was willing to consider a financial support request outside its usual funding cycle due to the emergent nature of the pandemic; members of the scientific committee also critiqued the protocol and raised specific concerns about the safety of HCQ in individuals who develop COVID-19. This reflected reports of cardiac pathology in hospitalized patients with COVID-19 [43] as well as potential issues with arrhythmogenic drugs in individuals infected with SARS-CoV-2 [44, 45]. Accordingly, two cardiologists were added to the research team; both had extensive clinical trial experience, one specifically in remote monitoring of electrocardiograms (ECGs). A research coordinator also with extensive clinical trial experience was hired for the study; an internal medicine resident physician and a family practice resident physician subsequently volunteered to assist with clinical trial enrolment and monitoring.

### Addressing “Safety First”

The local institutional review board also proved responsive in the rapid turnaround of protocol and consent form submissions. A major IRB concern was the safety of study participants and research staff; person-to-person contact and/or in-person visits to hospital facilities was discouraged. This had been anticipated and was the basis of several decisions: (1) All recruitment and enrolment was to be done via telephone and/or e-mail without any physical contact; (2) study drug would be delivered to participants’ home addresses; (3) the pre- and on-study testing for coronavirus SARS-CoV-2 would be done via a self-administered test in the participants’ homes; (4) ECG monitoring for possible cardiotoxicity such as QT interval prolongation would be accomplished by delivering a remote smartphone attachment to participant home addresses, downloading the application software from the Internet, and submitting tracings electronically. Having a cardiologist in the research group review participant ECGs on a daily basis was acceptable to the IRB as a safety measure. US Food and Drug Administration (FDA) guidance on the use of medical products during the COVID-19 public health emergency was followed in selecting testing and monitoring devices [46]. Smartphones had been used for remote ECG monitoring for a number of years [47] and there was an increasing use of this technique in clinical trials. Software using an attachment allowing 6-lead ECG monitoring had just come onto the market and this was selected over single-channel monitoring software [48]. It was noted that participants taking and uploading their own ECGs were likely to be unfamiliar with this technology with no opportunity for “hands on” training. Regarding SARS-CoV-2 testing, a newly released home saliva RNA PCR assay had received emergency use authorization (EUA) from the FDA and this was chosen as a less uncomfortable test that was easier to perform in the home setting than a nasal swab-based assay.

### Study outcomes

Between the start of the pandemic and May 1, 2020, the MLHS had had 801 total COVID-19 discharges; individual hospitals saw 121 to 323 discharges. The local rate of hospitalizations peaked in mid-April 2020 with 42 daily COVID-related admissions and then declined through the early fall despite rising numbers elsewhere in the USA (Table 1). By mid-September 2020, there were only 1–2 COVID-19 admissions daily. Study enrolment started in June 2020 and thus missed the local first peak of COVID-19 cases which occurred during the three organizational months.

Table 2 shows that between June and September 2020, nearly 500 household members potentially exposed to COVID-19-positive individuals were contacted via telephone or e-mail. Lack of a response to an attempted study contact was the major reason for missed enrolment. Overall, 54 individuals agreed to enroll in the study but 3 withdrew prior to randomization. Ultimately, 32 enrollees were assigned to the HCQ treatment arm but 7 withdrew prior to completion leaving 25. The other 19 were randomized to the observation arm but 2 withdrew prior to completion leaving 17.

**Table 2:**
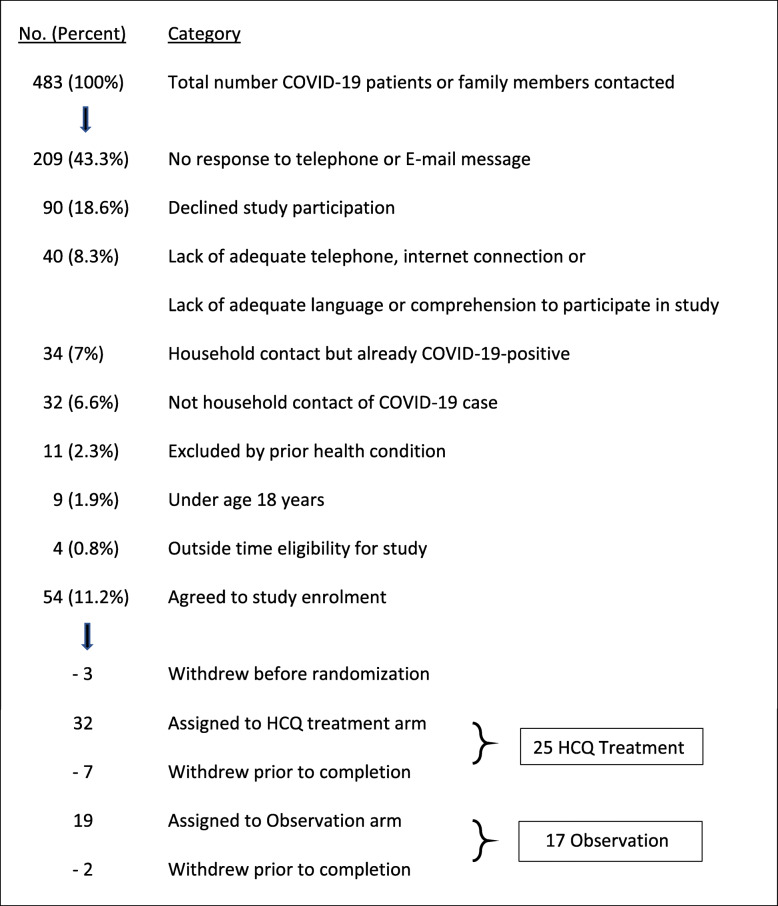
Enrolment, consent, and randomization for study

### Reliability of ECG data submission

**Fig. 1:**
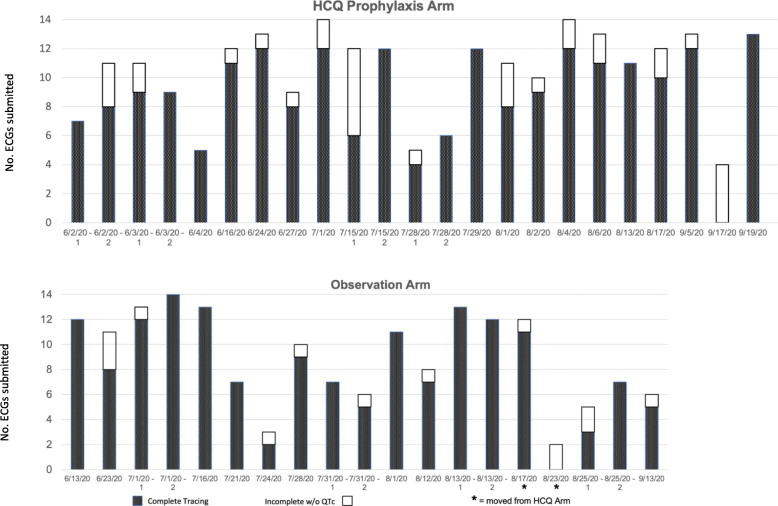
Number and adequacy of ECGs submitted by study participants. Columns depict tracings per individual listed by date of first ECG; the great majority of ECGs submitted were technically adequate (dark portion of bar) though QTc values could not always be determined (clear portion of bar)

Figure 1 shows the number of ECG recordings submitted by each study enrollee by date of first tracing. Two individuals assigned to the HCQ study arm declined to take the drug but agreed to do ECG monitoring and follow-up surveys; their data have been shown with the observation group. There was a wide range of compliance in submitting requested ECG tracings and some were of poor quality. It was not clear whether the technically inadequate tracings were related to individuals’ technical abilities or the quality of the transmission lines [1].

### Development of positive COVID-19 tests in HCQ-treated and observation groups

Of the 42 participants completing the study, 9 had positive SARS-CoV-2 saliva tests at baseline after randomization but before the start of the treatment/observation period; seven of these were symptomatic. This number was consistent with the high infectivity expected from COVID-19 but provided no information about the efficacy of HCQ in preventing infection. No individuals converted from SARS-CoV-2 negative to positive over the 14-day study interval so this also did not allow determination of the efficacy of HCQ in preventing acquisition of coronavirus. One individual in the HCQ treatment arm complained of a swollen lip but this was self-limited despite continued HCQ and judged unrelated to the study drug. No evidence of HCQ or viral cardiotoxicity was encountered.

## Discussion

### Efficacy of HCQ in preventing SARS-CoV-2 transmission

Study results did not allow conclusions about the efficacy of HCQ in preventing acquisition of SARS-CoV-2 infection via household contact with individuals diagnosed with COVID-19. Larger randomized and/or blinded studies of HCQ prophylaxis in COVID-19-exposed populations have been proposed [49] and published; one randomized, double-blinded, placebo-controlled study involving 821 US and Canadian participants was published in August 2020 [50] and an open-label, cluster-randomized trial covering 2314 healthy contacts of COVID-19 index cases in Spain was published in February 2021 [51]. These concluded that there was no significant difference in new illnesses consistent with COVID-19 in exposed individuals who received HCQ or placebo; side effects were more common with HCQ than placebo, but no serious adverse reactions were reported. Dosage regimens of HCQ differed in the two trials and subsequent correspondence suggested even larger trials might show differences reaching statistical significance [52]. However, another study published in early 2021 involved 671 households from the USA and showed no difference in COVID-19 disease acquisition in the HCQ versus vitamin C comparator groups but a higher adverse event rate in the HCQ group [53]. The EUA for HCQ use in the treatment of COVID-19 in the USA had been revoked on June 15, 2020 [54]. A systematic review and meta-analysis of randomized trials of HCQ for COVID-19 prophylaxis published in January 2021 concluded that there was no clinical benefit [55].

### Lessons from conducting a HCQ prophylaxis trial early in the COVID-19 pandemic

The COVID-19 pandemic evolved over a matter of months; concern in the USA was tempered at first by recollections of past outbreaks of SARS and MERS that were deadly but largely spared the USA. Once the danger was clearer with cases developing locally, there was a tremendous amount of news about COVID-19, mostly preliminary, incomplete, and sometimes incorrect and alarmist. In addition to the work caring for infected individuals and protecting staff and the public, healthcare practitioners were hampered by the lack of a rapid and accurate diagnostic test for SARS-CoV-2. The concept of a research trial of HCQ in preventing COVID-19 after household exposure was straightforward but obtaining consumer input was problematic. Most individuals were avoiding hospitals and physicians’ offices and videoconferencing was in limited use. Some MLHS physicians did speak with longstanding patients about COVID-19 studies during other interactions during the early pandemic. Feedback was positive about access to COVID-19 studies and developing information locally but there was no opportunity for a group advisory meeting or survey.

The lack of HCQ, a previously widely available drug, was surprising but should not have been unexpected. Speculation about HCQ in treating or preventing COVID-19 was common in the news in spring/summer 2020; despite a lack of human trial data, a vigorous and oftentimes acrimonious debate engulfed healthcare agencies and the highest levels of government. Practical implications of this argument about HCQ’s role in managing COVID-19 led to the study group being careful not to imply any proven benefit from HCQ and employ extensive precautions to avoid HCQ side effects especially cardiotoxicity.

COVID-19 infection in a member of the research group was a stark reminder of the risks of studying an infection in the midst of a pandemic. Given rising infection rates locally, it was always a statistical possibility. The impact was minimized by having a moderate-sized group of researchers with some redundancy in specialties. Lack of further cases validated the “no person-to-person contact” approach to study planning and execution.

Enrolment in the clinical trial largely mirrored the number of new cases and hospital admissions in our locality despite rising numbers elsewhere. There were fewer opportunities to recruit participants when COVID-19 hospitalizations fell to a low level in late summer 2020. The debate over HCQ use was another factor as data about the lack of benefit of HCQ against COVID-19 was reaching the public by that time. More infected individuals and/or their household contacts were declining to consider study enrolment by September. Also, several individuals out of the 54 who initially consented to participate changed their minds, withdrew after randomization (7 out of 32 assigned to the HCQ group versus 2 of 19 from the observation group), or declined to take study medication though complying with the monitoring protocol. There likely would have been more participants enrolled if follow-up calls had been made to the households that did not initially respond. However, limitations in time available and investigator effort did not allow this; there was also an ethical consideration regarding how hard to push for enrolment during a pandemic.

The local clinical research situation would have benefited from better pre-pandemic coordination and planning as the MLHS health system was isolated in some ways. The local university, of which MLHS is an academic affiliate, was occupied with clinical crisis management and then its own research programs. It would have been ideal to be part of a network of community institutions cooperating on outbreak reporting, research opportunities, and resource sharing. This could have allowed our study to continue recruitment in areas seeing a high incidence of COVID-19. However, there is no organized system in the USA similar to the UK RECOVERY project [56]. Even prior ad hoc attempts to conduct studies across systems within the state have been hindered by differing paperwork requirements, lack of ability to share funding, etc. Thus, despite numerous COVID-19 cases and often individually excellent facilities, the search for effective drugs against coronavirus SARS-CoV-2 in the USA has been termed uncoordinated and studies underpowered [57].

## Conclusions

1. Timing and flexibility in the study design were essential. From the beginning, it was expected and hoped that the increasing COVID-19 case counts would come under control at some point. Rapid organization and study implementation was thus critical to enrolment. In place of large investigator group meetings, serial reviews of the study protocol and proposed implementation steps allowed everyone to comment remotely and with greater safety.
2. Multidisciplinary composition of the research group proved to be important, perhaps more so than in other study types more narrowly focused in location or type of recruitment/data collection. For example, infectious disease physicians were versed in infection control during pandemics; a hospital component facilitated early identification of potential study households and permission to contact possible enrollees. Expertise in pharmacy services, a longstanding liaison with an institutional review board, experience in ECG remote monitoring and data interpretation, and a veteran clinical study coordinator covered many areas of need. Only the addition of researchers experienced in remote ECG monitoring was needed when this issue came up during safety discussions.
3. Cooperation among multiple groups such as study investigators, the hospital pharmacy, funding sources, and the health system institutional review board was critical to quickly writing and implementing the research protocol. Rapid responses to queries and a willingness to “think outside the box” allowed several barriers to be overcome including traditional funding and review deadlines.
4. Innovation with new tests and devices allowed the trial to be conducted safely during a pandemic; without newly introduced home diagnostic SARS-CoV-2 test kits and remote ECG monitoring hardware and apps, the study would not have been feasible for a small group with limited resources. Even new terminology was involved with the use of “no face-to-face personal exposure” or “no physical contact” becoming the preferred terms over “no in-person contact” or “no personal contact” as it was pointed out that videoconferencing is a form of in-person/personal contact. Openness to a remote consent process, prioritizing schedules for third-party delivery of study materials, and electronically assisting participants in collection and transmission of ECG tracings without prior extensive training were also important.

Some things that could have been improved and/or need to be addressed in the future were:

(5) Exclusion from the trial of individuals who lacked telephone and Internet connectivity needed to participate was a concern; this affected up to 8.3% of households contacted. The barrier includes comprehension and language issues as well as strictly technical limitations. Accordingly, populations such as lower income individuals, immigrants, those living in rural areas, and the elderly could be negatively affected, as has been seen during COVID-19 vaccine rollouts. Provision of cell phone or other Internet access as part of a study, ideally with translation services available, could be explored.
(6) Better coordination among local health systems in the conduct of clinical studies could have increased recruitment and shortened study time with more hospitals participating. Unfortunately, such advance coordination is uncommon outside large academic health systems in the USA. Even standardizing paperwork such as consent forms across systems can be extremely difficult. A regional or national effort such as RECOVERY in the UK has been suggested as a model for gathering data quickly in an outbreak or other emergency.

## Data Availability

Data sets generated and/or analyzed during the clinical study are not publicly available but are available from the corresponding author upon reasonable request.

## Abbreviations

ECG: Electrocardiogram
EUA: Emergency use authorization
US FDA: United States Food and Drug Administration
MLHS: Main Line Health System
SSRF: Sharpe-Strumia Research Foundation
